# Structure determination of 3*H*-benzimidazol-1-ium chloride monohydrate

**DOI:** 10.1107/S2056989026008236

**Published:** 2026-08-14

**Authors:** Mattia Lopresti

**Affiliations:** a Department of Science and Technological Innovation, University of studies of the Eastern Piedmont "Amedeo Avogadro", viale Teresa Michel 11, 15152, Alessandria, Italy

**Keywords:** crystal structure, hydrogen bonding, single-crystal X-ray diffraction, benzimidazolium salt

## Abstract

The title compound, C_7_H_7_N_2_^+^·Cl^−^·H_2_O, crystallizes in the ortho­rhom­bic space group *Pnma*. The organic mol­ecule lies on a crystallographic mirror plane, resulting in an asymmetric unit comprising one-half of a benzimidazolium cation. The crystal packing features N—H⋯Cl, O—H⋯O, and O—H⋯Cl hydrogen bonds, which connect the ionic and neutral components into a three-dimensional supra­molecular framework. The compound was obtained serendipitously during investigations of reactions involving NbCl_5_ and *o*-phenyl­enedi­amine.

## Abstract 2

The title benzimidazolium chloride hydrate crystallizes in the ortho­rhom­bic space group *Pnma*. It was isolated as an unexpected product from a reaction mixture containing NbCl_5_ and *o*-phenyl­enedi­amine.

## 1. Chemical context

**Figure 1:**
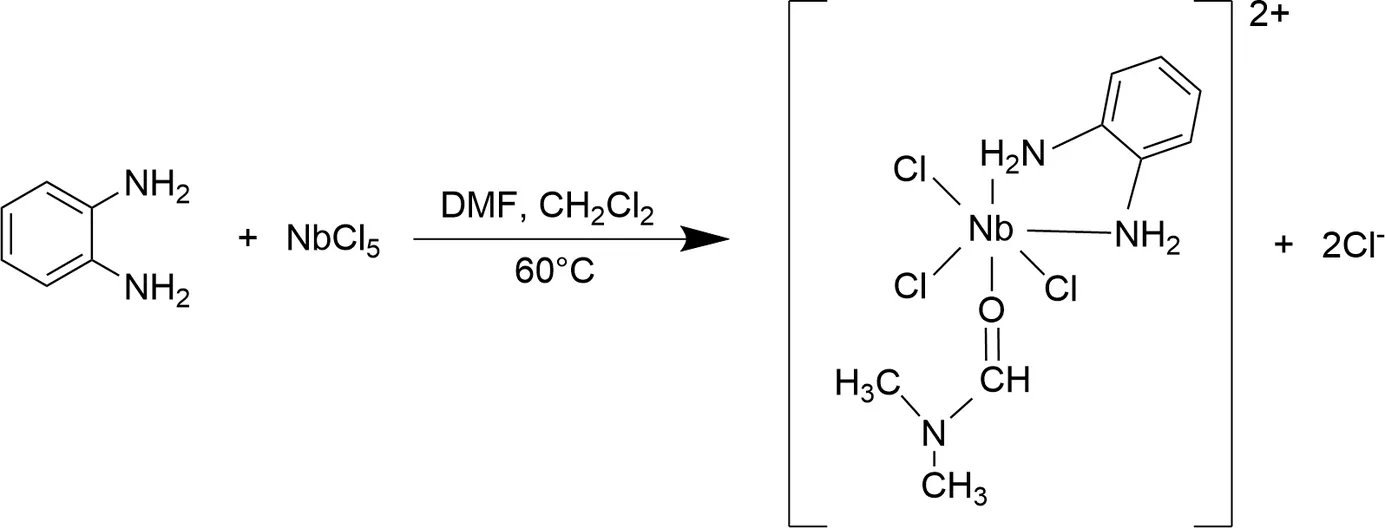
*CHEMDRAW* scheme of the expected reaction.

The title compound was identified during attempts to prepare a niobium(V) metal–organic complex for catalytic applications (Fig. 1 ▸) from NbCl_5_ and *o*-phenyl­enedi­amine in a CH_2_Cl_2_/DMF mixture (Drzeżdżon *et al.*, 2025*a* ▸,*b* ▸). Instead of the expected niobium-containing complex, the reaction afforded a heterogeneous precipitate composed of particles with different colours and sizes. X-ray powder diffraction (XRPD) suggested the presence of the title compound tog­ether with an additional amorphous component. A portion of the precipitate was dispersed in water, and an intense orange solution and a white insoluble solid were obtained (Fig. S1 in the supporting information). The insoluble fraction, separated by filtration, was characterized by XRPD and found to be amorphous, whereas slow evaporation of the orange solution yielded single crystals suitable for X-ray diffraction analysis (Figs. S2 and S3 in the supporting information).

The role of niobium-containing species in the formation of the title compound cannot be established from the present experiments. Niobium-containing oxides have been reported to promote cyclization reactions leading to benzimidazole derivatives under suitable conditions (Deepak *et al.*, 2025 ▸), whereas related benzimidazole-forming condensations are also known to proceed under catalyst-free conditions (Vlocskó *et al.*, 2024 ▸). Moreover, NbCl_5_ readily undergoes hydrolysis in the presence of water, producing niobium oxo species and hydro­chloric acid. Consequently, the available experimental evidence does not allow the participation of niobium-containing species in the formation of the title compound to be assessed, and no mechanistic inter­pretation is proposed.

The benzimidazole scaffold is a common structural motif in numerous functional derivatives, particularly inter­esting in medicinal chemistry (Tahlan *et al.*, 2019 ▸). The title compound corresponds to its protonated form, and the determination of its crystal structure provides reference structural data for the parent benzimidazolium framework and its supra­molecular behaviour in the solid state, facilitating comparison with related benzimidazole-based compounds.
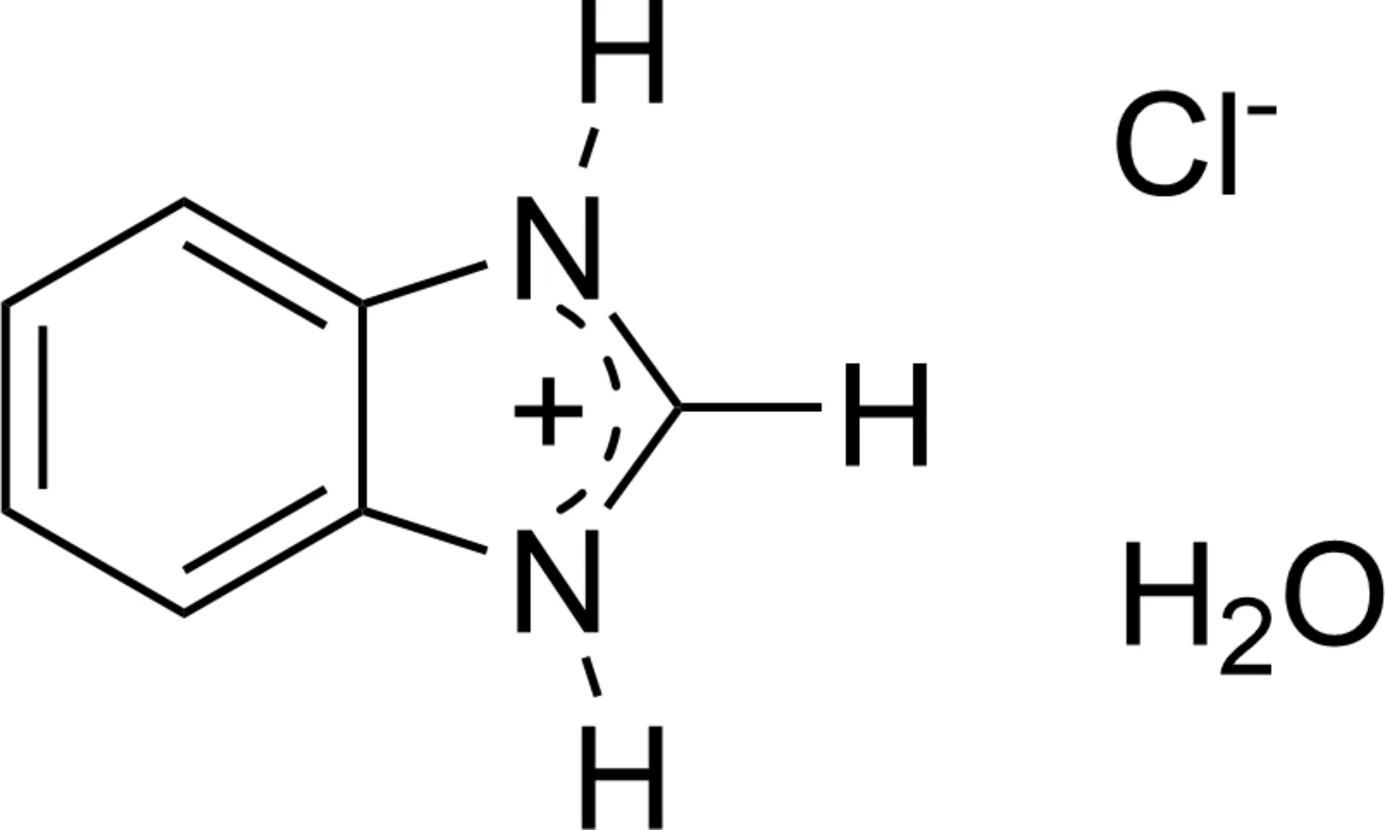


## 2. Structural commentary

**Figure 2:**
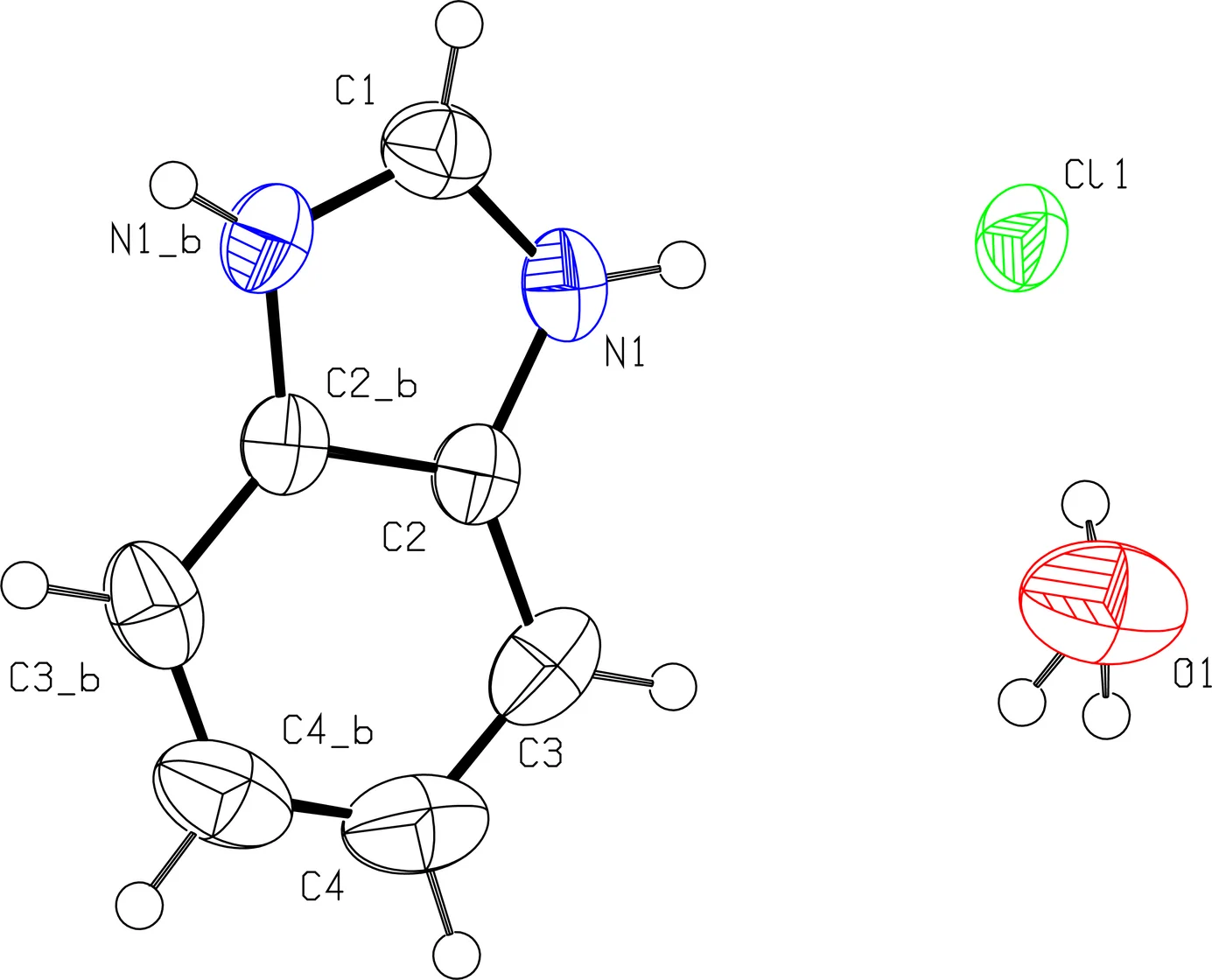
The asymmetric unit of the title compound, showing the naming scheme. The displacement ellipsoids are shown at the 50% probability level. Symmetry code: (b) *x*, −*y* + 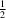, *z*.

The title compound (Fig. 2 ▸) crystallizes in the ortho­rhom­bic space group *Pnma*, with the asymmetric unit comprising one half of a 3*H*-benzimidazol-1-ium cation, one chloride anion and one water mol­ecule. The complete cation is generated by crystallographic mirror symmetry, with only the apical carbon atom C1 of the imidazolium ring lying on the mirror plane. The mol­ecular geometry is otherwise unexceptional and comparable to related benzimidazole derivatives reported in the literature. The remainder of the benzimidazolium framework is completed by reflection, resulting in a planar heteroaromatic system in the solid state.

Both nitro­gen atoms of the benzimidazolium cation are protonated (charge-assisted tautomeric protonation) consistent with a delocalized imidazolium-type cation giving an overall +1 charge that is balanced by the chloride anion. The chloride ion and the water mol­ecule also reside on crystallographic mirror planes, leading to a highly symmetric arrangement of the ionic and neutral components within the unit cell. This symmetry imposes geometric constraints on the hydrogen-bonding environment and results in equivalent N—H donor sites related by crystallographic symmetry.

Overall, the crystal structure is consolidated by a network of N—H⋯Cl and O—H⋯Cl hydrogen bonds (Table 1 ▸) involving the chloride anion and water mol­ecule, which link the ionic and neutral components into an extended supra­molecular arrangement.

## 3. Supra­molecular features

**Figure 3:**
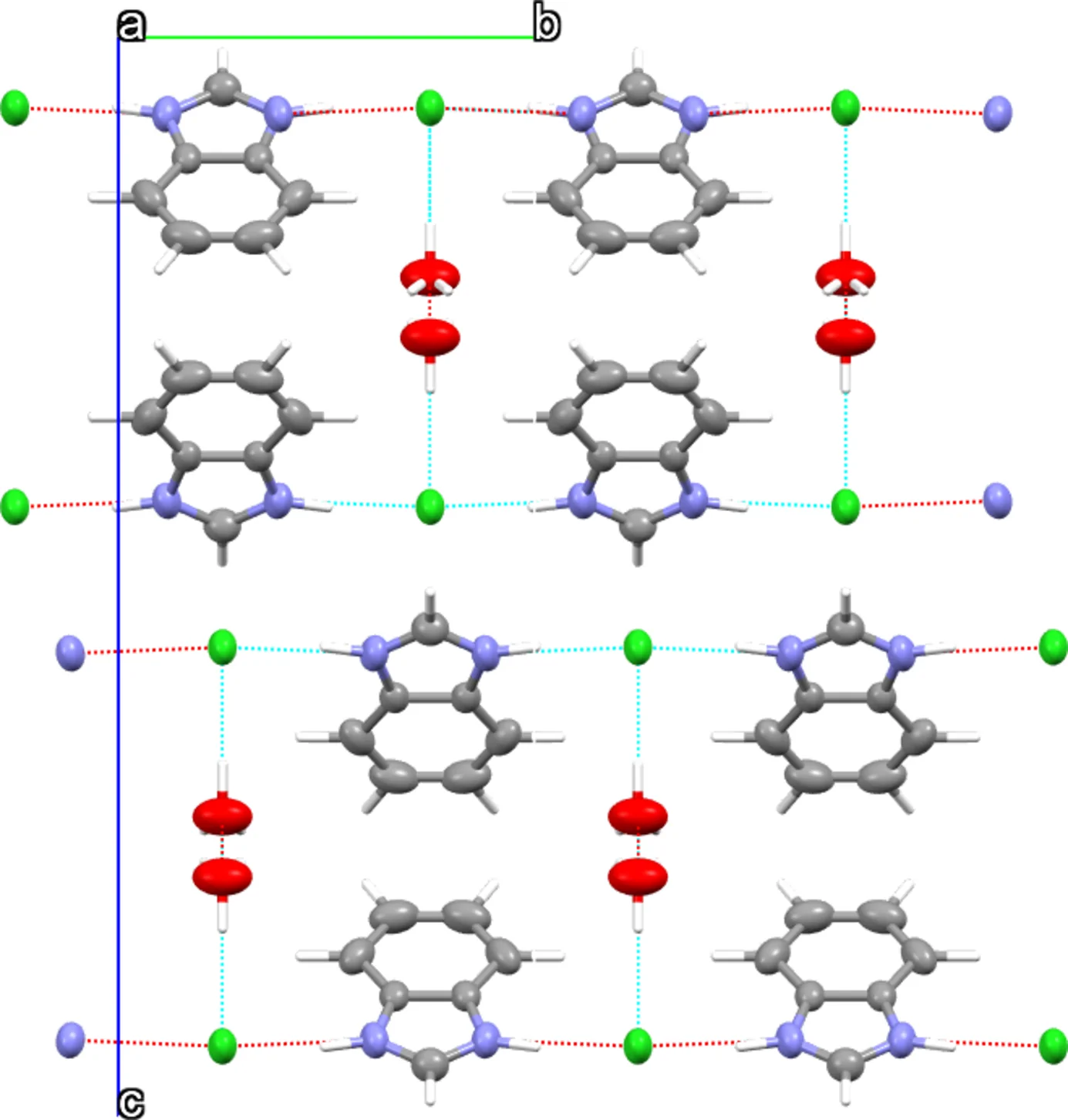
Packing of the title compound along the *a* axis.

The crystal structure is dominated by a hydrogen-bonding network involving and O—H⋯Cl, O—H⋯O, and N—H⋯Cl inter­actions (Table 1 ▸, Figs. 3 ▸ and 4 ▸). The primary structural motif consists of hydrogen-bonded water mol­ecules and chloride anions arranged in parallel one-dimensional arrays extending along the crystallographic *a* axis.

Within such chains, each water mol­ecule acts as a bifurcated hydrogen-bond donor, engaging in two distinct inter­actions: one O—H⋯Cl hydrogen bond directed towards a chloride anion and a second O—H⋯O inter­action linking adjacent water mol­ecules into a zigzag chain along the [100] direction.

The benzimidazolium cations bridge neighbouring chains through nearly linear N—H⋯Cl hydrogen bonds, thereby consolidating the crystal into a three-dimensional hydrogen-bonded framework. The angle defined between the N—H⋯Cl and O—H⋯Cl inter­actions is close to orthogonal, being 87.5 (2)°.

The chloride ions form chains running parallel to the water arrays and are linked to them via O—H⋯Cl hydrogen bonds, resulting in a set of alternating, closely associated anionic and neutral filaments. The topology of these inter­actions gives rise to planar supra­molecular columns defined by the combined water–chloride network.

Besides the classical hydrogen bonds, the crystal packing is reinforced by several short inter­molecular contacts. The chloride anion lies above the centroid of the imidazolium ring [Cl⋯centroid = 3.518 (3) Å, N1—centroid⋯Cl = 86.2 (8)°], consistent with a weak anion⋯π inter­action involving the positively charged heteroaromatic ring. Moreover, the hydrogen atom bonded to the C1 atom of the imidazolium ring is directed towards the chloride anion, giving a nearly linear C—H⋯Cl contact. Finally, neighbouring benzimidazolium cations are connected through weak C—H⋯π inter­actions involving the aromatic H4 atom and the centroid of a symmetry-related benzene ring (H⋯centroid = 3.33 Å).

## 4. Database survey

A search of the Cambridge Structural Database (CSD, Version 2026.1; Groom *et al.*, 2016 ▸) was performed using both unit-cell and structural similarity searches. No entries corresponding to *3H*-benzimidazol-*1*-ium chloride monohydrate were found, indicating that the crystal structure of the title compound has not previously been reported. The structural similarity search retrieved several benzimidazole derivatives containing closely related mol­ecular fragments, including POLMIO (Tamasi *et al.*, 2015 ▸) and CACGOD (Blake *et al.* 2001 ▸), while the unit-cell search identified structures crystallizing in similar unit cells, including CUWPUI (Sosa-Rivadeneyra *et al.*, 2020 ▸), DEVBAK (Lu *et al.*, 2018 ▸) and UVEHEJ (Zuev *et al.*, 2011 ▸).

Comparison of selected geometrical parameters confirms the close similarity of the benzimidazolium framework. The N1—C1 and N1—C2 bond lengths are 1.313 (3) and 1.382 (3) Å, respectively, in the title compound, compared with 1.324 (5)/1.389 (4) Å for POLMIO and 1.325 (3)/1.395 (3) Å for CACGOD, while the C2—C2′ bond length within the imidazolium ring is 1.380 (4) Å, compared with 1.386 (4) and 1.391 (3) Å, respectively. Likewise, the N—H⋯Cl hydrogen bond displays a comparable donor–acceptor distance to that observed in POLMIO [N⋯Cl = 3.087 (2) and 3.073 (3) Å, respectively].

Powder X-ray diffraction measurements were carried out on the original heterogeneous precipitate prior to its dispersion in water. Qualitative phase analysis performed with *DIFFRAC·EVA* (Bruker AXS GmbH, Karlsruhe, Germany) using the ICDD PDF-2 database showed good agreement with PDF card 17-0999 (Nielsen, F.; Private Communication, Monsanto Research Corporation, Dayton, OH, USA) assigned to benzimidazolium chloride hydrate (Figs. S4 and S5 in the supporting information). Following the single-crystal structure determination, the experimental powder diffraction pattern was refined by the Rietveld method using the structural model reported here. The refinement accurately reproduces all crystalline reflections (*R*_p_ = 2.518, *R*_wp_ = 3.285; Fig. S6 in the supporting information), while the remaining broad scattering is attributed to an amorphous component present in the original precipitate. These results demonstrate that the crystalline phase present in the original precipitate corresponds to the title compound, whereas the remaining material is amorphous, with no evidence of an additional crystalline polymorph.

## 5. Synthesis and crystallization

NbCl_5_ (1mmol) was dissolved in 10 mL of a 1:1 (*v*/*v*) CH_2_Cl_2_/DMF mixture under a nitro­gen atmosphere. *o*-Phenyl­enedi­amine (1 mmol) was then added, and the reaction mixture was heated at 333 K under stirring for 2h until the initially yellow suspension became a clear orange solution. While still hot, 10 mL of water were added, resulting in the formation of a brick-red precipitate. The solid was collected by filtration and dried in the oven at 333 K overnight.

Optical microscopy revealed that the precipitate consisted of particles with different shades and sizes, approximately 100–200 µm (Fig. S1 in the supporting information). X-ray powder diffraction analysis indicated the presence of both crystalline and amorphous components. The material exhibited slight hygroscopic behaviour upon exposure to air, becoming darker and more compact after grinding. To avoid possible structural changes associated with grinding, a small portion of the original precipitate was instead dispersed in water. This treatment produced an intense orange solution together with a white insoluble solid (Fig. S1 in the supporting information). The latter was separated by filtration, dried in air overnight and found to be amorphous by X-ray powder diffraction.

The orange solution was left to evaporate slowly at room temperature. Orange single crystals suitable for single-crystal X-ray diffraction analysis were obtained after 5 days (Fig. S2 in the supporting information). Following structure determination, Rietveld refinement of the powder X-ray diffraction pattern of the original precipitate showed that all crystalline reflections are accounted for by the crystal structure reported in the present paper, whereas the remaining broad scattering arises from an amorphous phase (Fig. S4 in the supporting information).

## 6. Refinement

Crystal data, data collection and structure refinement details are summarized in Table 2 ▸. The N-bound H atom and the H atom on C1 were freely refined. The C-bound H atoms and the water H atoms were positioned geometrically (C—H = 0.93, O—H = 0.84–0.85 Å) and refined as riding with *U*_iso_(H) = 1.2*U*_eq_(C) or 1.5*U*_eq_(O).

## Supplementary Material

Crystal structure: contains datablock(s) I. DOI: 10.1107/S2056989026008236/meu2004sup1.cif

ESI file containing optical microscope photos and XRPD refinement. DOI: 10.1107/S2056989026008236/meu2004sup3.docx

Supporting information file. DOI: 10.1107/S2056989026008236/meu2004Isup3.cml

CCDC reference: 2562397

Additional supporting information:  crystallographic information; 3D view; checkCIF report

## Figures and Tables

**Figure 4:**
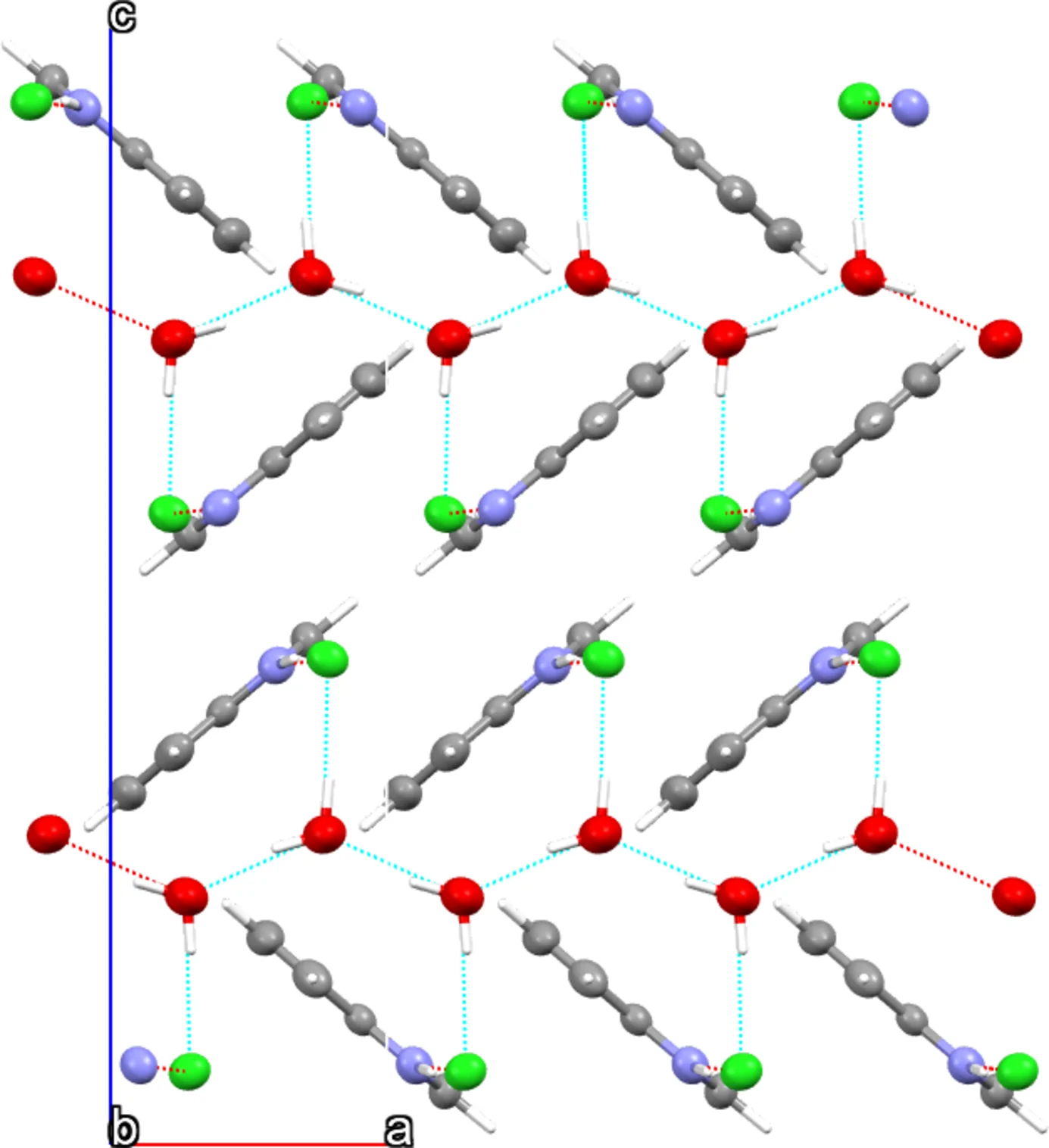
Packing of the title compound along the *b* axis.

**Table 1:** Hydrogen-bond geometry (Å, °)

*D*—H⋯*A*	*D*—H	H⋯*A*	*D*⋯*A*	*D*—H⋯*A*
N1—H2⋯Cl1	0.82 (3)	2.27 (3)	3.087 (2)	172 (3)
O1—H5⋯Cl1	0.84	2.44	3.272 (3)	176
O1—H6⋯O1^i^	0.85	2.06	2.827 (4)	150
C1—H1⋯Cl1^ii^	0.87 (4)	2.62 (3)	3.485 (4)	176 (3)

Symmetry codes: (i) 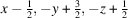; (ii) 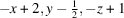.

**Table 2:** Experimental details

Crystal data	
Chemical formula	C_7_H_7_N_2_^+^·Cl^−^·H_2_O
*M*_r_	172.61
Crystal system, space group	Orthorhombic, *Pnma*
Temperature (K)	298
*a*, *b*, *c* (Å)	5.1532 (8), 8.0247 (10), 20.833 (3)
*V* (Å^3^)	861.5 (2)
*Z*	4
Radiation type	Mo *K*α
μ (mm^−1^)	0.39
Crystal size (mm)	0.10 × 0.05 × 0.02

Data collection	
Diffractometer	Xcalibur, Sapphire3
Absorption correction	Multi-scan (*CrysAlis PRO*; (Rigaku OD, 2022 ▸)
*T*_min_, *T*_max_	0.500, 1.000
No. of measured, independent and observed [*I* > 2σ(*I*)] reflections	2272, 1045, 673
*R*_int_	0.033
(sin θ/λ)_max_ (Å^−1^)	0.686

Refinement	
*R*[*F*^2^ > 2σ(*F*^2^)], *wR*(*F*^2^), *S*	0.051, 0.123, 1.03
No. of reflections	1045
No. of parameters	65
H-atom treatment	H atoms treated by a mixture of independent and constrained refinement
Δρ_max_, Δρ_min_ (e Å^−3^)	0.22, −0.29

Computer programs: *CrysAlis PRO* (Rigaku OD, 2022 ▸), *SHELXT2018/2* (Sheldrick, 2015*a* ▸), *SHELXL2018/3* (Sheldrick, 2015*b* ▸), *ORTEP-3* (Farrugia, 2012 ▸) and *Mercury* (Macrae *et al.*, 2020 ▸).

## supplementary crystallographic information

### 3H-Benzimidazol-1-ium chloride monohydrate Crystal data

**Table:**

C_7_H_7_N_2_^+^·Cl^−^·H_2_O	*D*_x_ = 1.331 Mg m^−^^3^
*M**_r_* = 172.61	Mo *K*α radiation, λ = 0.71073 Å
Orthorhombic, *P**n**m**a*	Cell parameters from 786 reflections
*a* = 5.1532 (8) Å	θ = 3.9–26.4°
*b* = 8.0247 (10) Å	µ = 0.39 mm^−^^1^
*c* = 20.833 (3) Å	*T* = 298 K
*V* = 861.5 (2) Å^3^	Rect. Prism, orange
*Z* = 4	0.10 × 0.05 × 0.02 mm
*F*(000) = 360	

### 3H-Benzimidazol-1-ium chloride monohydrate Data collection

**Table:**

Xcalibur, Sapphire3 diffractometer	1045 independent reflections
Radiation source: fine-focus sealed X-ray tube, Enhance (Mo) X-ray Source	673 reflections with *I* > 2σ(*I*)
Graphite monochromator	*R*_int_ = 0.033
Detector resolution: 8.6225 pixels mm^-1^	θ_max_ = 29.2°, θ_min_ = 3.9°
ω scans	*h* = −6→5
Absorption correction: multi-scan (CrysAlisPro; (Rigaku OD, 2022)	*k* = −10→8
*T*_min_ = 0.500, *T*_max_ = 1.000	*l* = −26→12
2272 measured reflections	

### 3H-Benzimidazol-1-ium chloride monohydrate Refinement

**Table:**

Refinement on *F*^2^	Primary atom site location: dual
Least-squares matrix: full	Hydrogen site location: mixed
*R*[*F*^2^ > 2σ(*F*^2^)] = 0.051	H atoms treated by a mixture of independent and constrained refinement
*wR*(*F*^2^) = 0.123	*w* = 1/[σ^2^(*F*_o_^2^) + (0.0557*P*)^2^] where *P* = (*F*_o_^2^ + 2*F*_c_^2^)/3
*S* = 1.03	(Δ/σ)_max_ < 0.001
1045 reflections	Δρ_max_ = 0.22 e Å^−^^3^
65 parameters	Δρ_min_ = −0.29 e Å^−^^3^
0 restraints	

### 3H-Benzimidazol-1-ium chloride monohydrate Special details

**Table:**

Geometry. All esds (except the esd in the dihedral angle between two l.s. planes) are estimated using the full covariance matrix. The cell esds are taken into account individually in the estimation of esds in distances, angles and torsion angles; correlations between esds in cell parameters are only used when they are defined by crystal symmetry. An approximate (isotropic) treatment of cell esds is used for estimating esds involving l.s. planes.

### 3H-Benzimidazol-1-ium chloride monohydrate Fractional atomic coordinates and isotropic or equivalent isotropic displacement parameters (Å^2^)

**Table:**

	*x*	*y*	*z*	*U*_iso_*/*U*_eq_	Occ. (<1)
Cl1	0.78656 (18)	0.750000	0.43495 (4)	0.0489 (3)	
O1	0.7747 (6)	0.750000	0.27793 (14)	0.0887 (11)	
H6	0.625537	0.785409	0.267147	0.133*	0.5
H5	0.786682	0.749999	0.318058	0.133*	
N1	0.6018 (4)	0.3844 (3)	0.42934 (10)	0.0449 (6)	
H2	0.653 (6)	0.480 (4)	0.4347 (15)	0.084 (11)*	
C1	0.7134 (8)	0.250000	0.45240 (18)	0.0478 (9)	
H1	0.843 (7)	0.250000	0.4788 (16)	0.046 (10)*	
C2	0.4052 (4)	0.3360 (3)	0.38826 (10)	0.0371 (5)	
C3	0.2311 (5)	0.4263 (4)	0.35142 (12)	0.0542 (7)	
H3	0.230102	0.542203	0.351544	0.065*	
C4	0.0611 (6)	0.3366 (4)	0.31492 (12)	0.0664 (9)	
H4	−0.058001	0.392979	0.289345	0.080*	

### 3H-Benzimidazol-1-ium chloride monohydrate Atomic displacement parameters (Å^2^)

**Table:**

	*U*^11^	*U*^22^	*U*^33^	*U*^12^	*U*^13^	*U*^23^
Cl1	0.0640 (7)	0.0319 (5)	0.0507 (5)	0.000	−0.0052 (4)	0.000
O1	0.069 (2)	0.142 (3)	0.0555 (16)	0.000	0.0050 (18)	0.000
N1	0.0508 (14)	0.0327 (11)	0.0511 (12)	−0.0059 (9)	0.0047 (11)	−0.0035 (10)
C1	0.041 (2)	0.056 (2)	0.0468 (19)	0.000	−0.0015 (18)	0.000
C2	0.0383 (13)	0.0352 (11)	0.0378 (11)	0.0003 (10)	0.0078 (11)	0.0007 (10)
C3	0.0580 (17)	0.0506 (15)	0.0541 (14)	0.0145 (13)	0.0120 (14)	0.0140 (13)
C4	0.0521 (17)	0.101 (2)	0.0457 (13)	0.0120 (15)	−0.0015 (13)	0.0103 (14)

### 3H-Benzimidazol-1-ium chloride monohydrate Geometric parameters (Å, º)

**Table:**

O1—H6	0.8499	C2—C2^i^	1.380 (4)
O1—H5	0.8382	C2—C3	1.385 (3)
N1—H2	0.82 (3)	C3—H3	0.9300
N1—C1	1.313 (3)	C3—C4	1.365 (4)
N1—C2	1.382 (3)	C4—C4^i^	1.390 (6)
C1—H1	0.87 (3)	C4—H4	0.9300
			
H6—O1—H5	109.3	C2^i^—C2—N1	106.31 (12)
C1—N1—H2	125 (2)	C2^i^—C2—C3	121.55 (16)
C1—N1—C2	108.5 (2)	C2—C3—H3	121.7
C2—N1—H2	126 (2)	C4—C3—C2	116.6 (3)
N1^i^—C1—N1	110.4 (4)	C4—C3—H3	121.7
N1—C1—H1	124.81 (17)	C3—C4—C4^i^	121.81 (16)
N1^i^—C1—H1	124.81 (17)	C3—C4—H4	119.1
N1—C2—C3	132.1 (2)	C4^i^—C4—H4	119.1
			
N1—C2—C3—C4	179.1 (2)	C2—N1—C1—N1^i^	−0.3 (4)
C1—N1—C2—C2^i^	0.2 (2)	C2^i^—C2—C3—C4	−0.4 (3)
C1—N1—C2—C3	−179.4 (3)	C2—C3—C4—C4^i^	0.4 (3)

Symmetry code: (i) *x*, −*y*+1/2, *z*.

### 3H-Benzimidazol-1-ium chloride monohydrate Hydrogen-bond geometry (Å, º)

**Table:**

*D*—H···*A*	*D*—H	H···*A*	*D*···*A*	*D*—H···*A*
N1—H2···Cl1	0.82 (3)	2.27 (3)	3.087 (2)	172 (3)
O1—H5···Cl1	0.84	2.44	3.272 (3)	176
O1—H6···O1^ii^	0.85	2.06	2.827 (4)	150
C1—H1···Cl1^iii^	0.87 (4)	2.62 (3)	3.485 (4)	176 (3)

Symmetry codes: (ii) *x*−1/2, −*y*+3/2, −*z*+1/2; (iii) −*x*+2, *y*−1/2, −*z*+1.
