# Supplementary material for: Structure determination of 3H-benzimidazol-1-ium chloride monohydrate
Source: Acta Crystallogr E Crystallogr Commun. 2026 Aug 14;82(Pt 9):1012–5. doi: 10.1107/S2056989026008236 (PMC13548756; doi:10.1107/S2056989026008236)
Supplement: Supplementary file 1 [file e-82-01012-sup3.docx]

**The structure determination of 3*H*-benzoimidazol-1-ium chloride monohydrate**

**Mattia Lopresti**

Department of Science and Technological Innovation, University of studies of the, Eastern Piedmont "Amedeo Avogadro", viale Teresa Michel 11, 15152, Alessandria, Italy

Correspondence e-mail: mattia.lopresti@uniupo.it

|  |  |
| --- | --- |

Figure S1 – Optical images of the heterogenous reaction product of the synthesis (left) and the same product after dispersion in water (right). The solid fraction and the solution were separated by filtration.

|  |  |
| --- | --- |

Figure S2 – Optical images of the recrystallized title compound by slow evaporation of the solution in Figure S1 (right).

|  |  |
| --- | --- |

Figure S3 – Pictures collected by the single-crystal X-ray diffractometer of the analyzed crystal of the title compound.

Figure S4 – Qualitative phase analysis carried out by DIFFRAC.EVA with ICDD PDF2.

Figure S5 – A detail of the 17-0999 card (Nielsen, F. Monsanto Research Corporation).

Figure S6 – Rietveld refinement of the title compound on the original product XRPD data. The structural model presented in this paper perfectly models the reflections of the pattern (Rp=2.518, Rwp=3.285). The presence of the amorphous phase is evidenced by the shape of the background function.
